# Effect of Environmental Factors on Germination and Emergence of Invasive *Rumex confertus* in Central Europe

**DOI:** 10.1155/2015/170176

**Published:** 2015-07-02

**Authors:** Jeremi Kołodziejek, Jacek Patykowski

**Affiliations:** ^1^Department of Geobotany and Plant Ecology, Faculty of Biology and Environmental Protection, University of Łódź, Banacha 12/16, 90-237 Łódź, Poland; ^2^Department of Plant Physiology and Biochemistry, Faculty of Biology and Environmental Protection, University of Łódź, Banacha 12/16, 90-237 Łódź, Poland

## Abstract

*Rumex confertus* is a biennial species native to Eastern Europe and Asia, where it thrives on meadow-steppes and glades in forest-steppe. This species has increased its range rapidly within central Europe, yet its biology is not well understood, which has led to poorly timed management. Effects of temperature, light, sodium chloride (NaCl), hydrogen ion concentration (pH), potassium nitrate (KNO_3_), and polyethylene glycol 6000 on seed germination were examined. Seedling emergence was examined for seeds sown at different depths in sand-filled pots. Seeds of *R. confertus* were nondormant at maturity. The germination percentage and rate of germination were significantly higher in light than in darkness. Secondary dormancy was induced in these seeds by 12 weeks of dark incubation at 4°C. The seeds of *R. confertus* undergo a seasonal dormancy cycle with deep dormancy in winter and early spring and a low level of dormancy in early autumn. Germination decreased as soil salinity increased. NO_3_^−^ increased the percentage and rate of germination in the studied species. Decrease in seedling emergence from the seeds buried at >0.5 cm may be due to deficiency of light. From our experiments, we conclude that the weed *R. confertus* normally becomes established in vegetation gaps or due to disturbance of the uppermost soil layer during the growing season through the germination of seeds originating from a long-lived seed bank.

## 1. Introduction

Invasive species are considered one of the main threats for biodiversity because they dramatically affect the structure and functioning of ecosystems. Features that affect the capacity of allow plants to be rapid or efficient colonizers include wide environmental tolerance, high levels of phenotypic plasticity, high fecundity, effective dispersal, ability to germinate across a wide range of conditions, high relative growth rate, early phenology, high competitive ability, and genome duplication (see [1] and the references therein). Studies of the principles of environmental control of germination may aid in the interpretation and, if possible, prediction of the field behaviour of troublesome weeds [2].

Reproductive traits have been repeatedly recognized as important determinants of invasive species success [3, 4]. Invasive plants compete with native plants for nutrients, water, and sunlight [5]. Successful germination in a wide range of conditions increases the probability of naturalization and subsequent invasion [3]. Understanding seed characteristics and seedling establishment patterns is essential for the development of effective management strategies for invasive species [6].

The genus of* Rumex* (Polygonaceae) comprises nearly 200 species that are mostly perennials. The plants of this genus grow widely in wetlands and tolerate a wide range of soil conditions, but they are more common on acidic and low nutrient soils [7]. The requisites for seed germination of many species of* Rumex* have been studied for several decades. In the germination assays, the species differed clearly in their light and temperature requirements [8]. For example,* R. obtusifolius* seed germinated in light as well as in dark period [9, 10]. Higher percentage of* R. crispus* germination is usually obtained when the seeds are exposed to light [11]. Germination of both* R. obtusifolius* and* R. crispus* is also induced in darkness by alternating high and low temperatures [12, 13]. Taylorson and Hendricks [14] suggested that in* R. crispus* high-temperature promotion of germination is due to the stimulated action of a low level of preexisting Pfr in the seeds. Gambi [15] demonstrated experimentally that* R. acetosa* germinated in darkness at constant temperatures, but species such as* R. crispus*,* R. conglomeratus*,* R. longifolius*,* R. sanguineus*, and* R. hydrolapathum* required light for germination [16]. Seeds of closely related species,* R. acetosa* and* R. scutatus*, can germinate immediately after dispersal, and they are short-lived [8]. An annual dormancy cycle for buried seeds was shown for* R. obtusifolius*,* R. conglomeratus*,* R. maritimus*,* R. sanguineus*,* R. crispus*, and* R. palustris*. These seeds are shed in autumn, and they germinate mainly in spring in consecutive years [8, 17].

**Figure 1:**
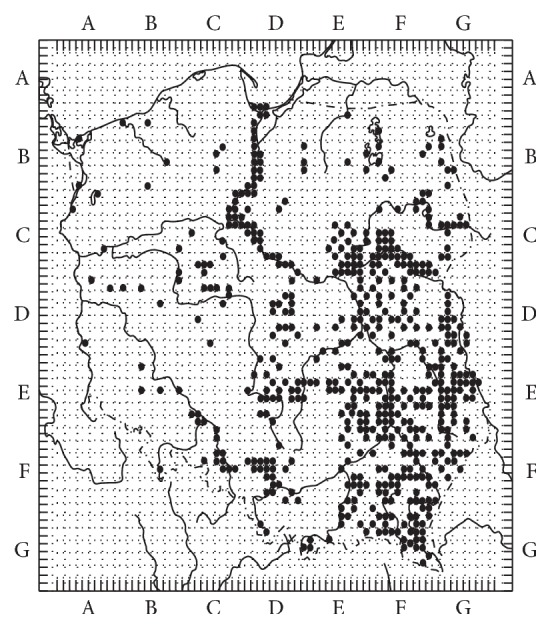
Distribution map of* Rumex confertus* in Poland using the ATPOL grid square system [18].

*Rumex confertus* Willd. (Asiatic dock) is a biennial species native to Eastern Europe and Asia, where it thrives on meadow-steppes and glades in forest-steppe [19, 20].* Rumex confertus* was found for the first time in Poland on the river Bug in 1873 and currently it appears a common plant in eastern Poland, reaching even the Baltic coast to the North, and it has many diffused localities also in western Poland (Figure 1) [18, 20, 21]. It is now widespread also in Baltic countries and treated as invasive plant in Lithuania [22], as well as Romania [23], Serbia [24], Bulgaria [24], Hungary [25], and Czech Republic [26]. Disturbed habitats (such as roadsides, railway tracks, and embankments) with good possibilities for fruit dispersal and establishment of seed are readily invaded, but the species also invades seminatural vegetation (e.g., meadows and riparian-scrub) [20, 27, 28].* Rumex confertus* spread in soils with potential large concentrations of heavy metals. Overall, traits exhibited by* R. confertus*, such as large amounts of seed production and high viability as well as rapid growth of the plants, have been repeatedly recognized as determinants of colonization success of invasive species on open areas [20, 26]. Fruits ripen between late June and mid-August, and seeds are dispersed between late August and October. The seeds (nutlets) are lustrous, red-brown, 2.7–3.2 × 1.8–2.3 mm, ovate-trullate or elliptic-rhombic [19, 26]. A single plant is capable of producing a large quantity of fruit and estimates range from 1550 to more than 4000 per individual [19]. The substantial fecundity and efficient dispersal of fruit by water, wind, and human-related factors contribute to its rapid spread. Like other seed traits, germination (the first step in colonizing a new habitat) can also evolve in response to the elective pressures of different habitats, thereby producing genetic differentiation [29]. In addition, it is still not clear if other traditional dormancy-braking methods can replace light and initiate germination; hence, a systematic study is required to investigate these areas.

The main objective of this study was to gather basic information on seed germination and seedling emergence of* R. confertus* to aid in controlling it. This study included examining (1) the effects of temperature, light, sodium chloride (NaCl), hydrogen ion concentration (pH), potassium nitrate (NO_3_^−^), and water stress on seed germination and (2) the effect of sand burial depth on seedling emergence. In addition, an attempt was made to evaluate the influence of potassium nitrate (NO_3_^−^) on early seedling development of* R. confertus*.

## 2. Materials and Methods

### 2.1. Seed Collection and Field Site Description

Freshly harvested mature nuts (hereafter referred to as seeds) were collected on 15th August 2014, from wild plants growing in natural habitat in the vicinity of Uniejów (51°96′N, 18°79′E), central Poland. This population is located along the banks of the river Warta in* Calthion palustris* community situated on an organic soil overlying sandy subsoil. Before sowing, the seed perianth was removed by rubbing them on a sieve and the seeds were cleaned by blowing [17].

The seeds were stored for 10 days in paper bags in darkness at room temperature and protected from humidity prior to the germination experiments or the stratification treatment. The seeds were stratified at 4°C in darkness for 10 weeks prior to the tests.

### 2.2. General Experimental Procedures

For each treatment, four replicates of 25 seeds were used; nontreated seeds were the control for each experiment. Unless otherwise noted the seeds were placed on wet filter papers (2 layers) in a 5 cm plastic Petri dishes. The filter paper was moistened initially with 3 mL of distilled water or the test solution. If necessary, 0.5–1 mL of the appropriate solution was added to maintain adequate moisture. Dishes were wrapped in a plastic film to reduce loss of water. The seeds were tested for germination in a 12 h photoperiod (Sylvania cool white fluorescent lamps, at 350 *μ*E m^−2^ s^−1^ intensity) and in darkness, which was achieved by wrapping Petri dishes in a double layer of aluminium foil.

Radicle protrusion was the criterion for germination. Unless otherwise noted, the germinated seed were counted and removed daily for a period of 20 days of any experiment. Counts in the dark treatments were made under dim green safe light. Germination percentages were determined on the basis of the number of viable seeds. The rate of germination was estimated using a modified Timson's index of germination velocity *G*/*t*, where *G* is seed germination percentage each day and *t* the total germination time [30]. Therefore if all of the seeds germinated in one day, the Timson's index would be 100 (i.e., 2300/23), and a higher value indicates more rapid germination.

### 2.3. Experiment 1: Germination Temperature Effects and Light/Dark Germination

These germination experiments were performed using fresh seeds (10 days after harvest). Four dishes of seeds were incubated in light at each of the following temperatures: 5°, 10°, and 22°C for 20 days. Another set of four dishes of seeds was tested for germination in darkness at each of the three temperature regimes. These temperatures approximated the mean daily maximum and minimum monthly temperatures in central Poland during the growing season: 4.8°C (early April and October), 10.3°C (late May), and 21.5°C (June and early July), when most seeds germinate in the natural habitat.

### 2.4. Experiment 2: Germination of Cold Stratified Seeds

Four 25-seed replicates were placed on the filter paper and placed in a refrigerator in darkness for 12 weeks at 4°C. This temperature is near-optimal for many seeds requiring low moisture and low temperature to break dormancy [31]. Germination conditions were the same as those described in Experiment 1 with fresh seeds.

### 2.5. Experiment 3: Germination Tests under Saline Conditions

To determinate the effect of salinity on germination of* R. confertus* freshly matured seeds, they were germinated in distilled water (control) and 10, 20, 40, 80, and 160 mM L^−1^ NaCl solutions. Four 25-seed replicates of each treatment were placed on filter paper in 3 cm Petri dishes with 3 mL of the test solution. The seeds were incubated at 22°C under light condition for 20 days. Preliminary experiment showed that constant 22°C temperature was optimal for germination of fresh seeds both in the light and in dark.

### 2.6. Experiment 4: Effect of Water Stress on Seed Germination

Due to the fact that this species is mostly found in moist environments [19, 20], a range of fairly moist conditions was simulated using polyethylene glycol 6000 (PEG-6000) solutions prepared according to Michel and Kaufmann [32]. Freshly matured seeds were incubated in 5 cm dishes on two sheets of filter paper moistened with 3 mL distilled water or the same volume of water solutions of PEG-6000 with osmotic potentials of −0.13, −0.25, −0.5, −0.75, and −1.0. The incubation conditions were identical to those described for Experiment 3. Germination of the seeds and the level of PEG-6000 solutions were assessed daily. Once a reduction in the level of PEG solution was detected, all filter papers and solutions were replaced with PEG-6000 solutions prepared on the day of replacement.

### 2.7. Experiment 5: Effect of pH on Seed Germination

Freshly matured seeds were exposed to buffer solutions (3 mL) of pH 4–9 in 1.0 increments. Distilled water (pH 5.7) was used as a control. Buffered pH solutions (2 mL) of 4.0, 5.0, and 6.0 contained (0.2 M) potassium hydrogen phosphate. 0.1 M sodium hydrogen phthalate buffer with pH 7.0 was used. For buffered solutions of 8.0 and 9.0, 0.2 M boric acid solution and 0.05 M borax solution were used. Final adjustments of each buffer were made using 0.1 M HCl or 0.1 M NaOH. The seeds were incubated under the conditions identical to those described for Experiment 3.

### 2.8. Experiment 6: Effect of NO_3_^−^ on Germination and on Radicle and Hypocotyl Lengths

To test the effects of NO_3_^−^ on germination and seedling growth, we used potassium nitrate (KNO_3_) chosen to represent the salt that may be present in the habitat along the banks of the river Warta. Freshly matured seeds were placed on filter paper in plastic Petri dishes wetted distilled water (control) and 0.5, 5, 10, 25, and 50 mM L^−1^ KNO_3_ (= NO_3_^−^) and incubated in a chamber at 22°C. Four Petri dishes were wrapped in aluminium foil (dark treatment) and four dishes were sealed with parafilm (light treatment) for 20 days. All solutions were monitored and maintained at pH 6.1. To test the effects of NO_3_^−^ on seedling growth germination tests were performed as described above and after twenty days, the seeds were no longer germinating, so all germinated seedlings were removed and their radicle and hypocotyl lengths were measured immediately.

### 2.9. Experiment 7: Examination of Seedling Emergence

Freshly matured seeds were planted at 0.5, 1.0, 1.5, 2, 2.5, 3, 3.5, and 4 cm depths in plastic pots (15-cm in diameter) filled with unsterilized sifted sand and incubated in a growth chamber at 22°C under 12 h light photoperiod. The pots were watered as needed to maintain adequate sand moisture. Emerged seedlings (cotyledons visible at the sand surface) were counted daily for 20 d and removed. The final percentage of emergence was defined as the percentage of seedlings emerged at the end of the experiment.

### 2.10. Data Analysis

Germination percentage was calculated as the number of germinated seeds to the total number of seeds tested. Germination data are expressed as means (±SD) of four replicates. One-way ANOVA was used to assess the effect of planting depth on emergence and seedling growth. Two-way ANOVA was used to determine the effect of each KNO_3_ treatment at each incubation light condition. Three-way ANOVA was used to determine the effects of temperature (*T*), light condition (*L*, light versus dark), and cold stratification (CS, nontreated versus 12-week cold stratified) on seed germination and rate of germination. Differences between means were considered to be significant at *P* < 0.05 by Tukey's multiple range test. Germination data were arcsine-transformed to ensure homogeneity of variance. All statistical tests were performed using a software package STATISTICA [33].

## 3. Results

### 3.1. Temperature and Light Requirements for Germination

**Figure 2:**
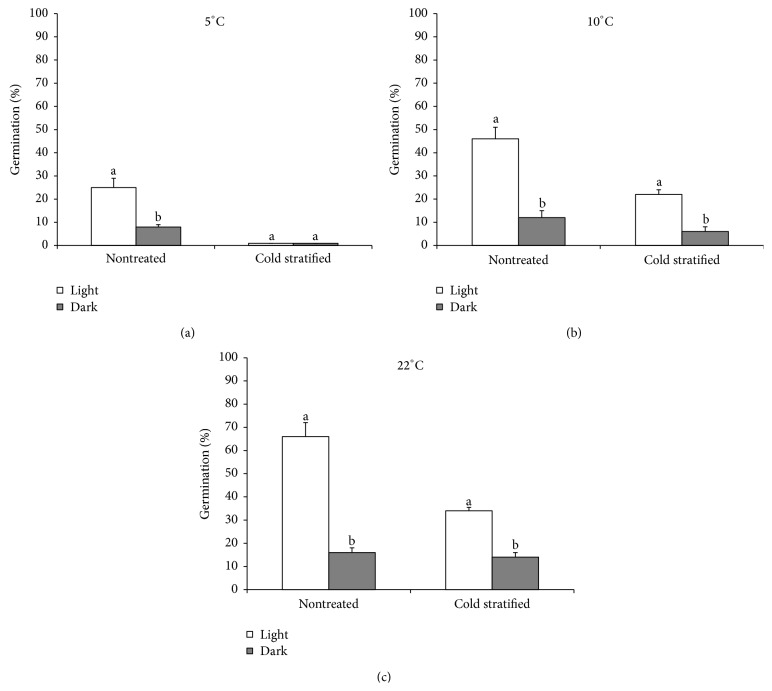
Effects of temperature and light condition on germination as recorded for nontreated and cold-stratified seeds of* Rumex confertus*. Different lower-case letters indicate significant differences in germination percentages of the same treatment between light and dark periods at 5°C (a), 10°C (b), and 22°C (c) for 20 days (Tukey's test at *P* < 0.05). Each value is a mean of four replicates of 25 seeds. Error bars indicating standard deviation.

Fresh seeds (i.e., after 10 days of dry storage) of* R. confertus* have fully developed embryos and are nondormant; they can germinate directly after dispersal. The seeds incubated in light germinated to much higher percentages than those incubated in darkness. Among the seeds incubated in light the germination was highest (66%) at 22°C and then dropped to 45% and 25% at 10°C and 5°C, respectively. Among the seeds incubated in darkness similar trend in germination was observed, namely, 22°C—16%, 10°C—12%, and 5°C—8% (Figures 2(a), 2(b), and 2(c)). Germination velocity (Timson's Index) was significantly higher when more light and higher temperatures were given (*P* < 0.001 for the difference). A three-way ANOVA showed that germination velocity and percentage of seed germination were significantly affected by light conditions (*P* < 0.001), temperature (*P* < 0.001), and their interaction (*P* < 0.001, Table 1).

Cold stratification had negative impact on* R. confertus* seed germination (Figures 2(a), 2(b), and 2(c)). Overall, the fresh seeds germinated better than the stratified ones: for example, cumulative germination of the nontreated seeds was 66% at 22°C in light, whereas only 34% after 12 weeks' stratification at this regime (Figure 2(c)). In addition, differences between germination in light and dark period were smaller after stratification than in the seeds tested after 10 days of dry-storage. This was due to reduced germination in both light and dark period under all the temperatures used. The rate of germination in both light and dark periods gradually diminished with temperature decrease. A three-way analysis of variance of germination indicated significant effects of light (*P* < 0.001), temperature (*P* < 0.001), cold stratification (*P* < 0.001), and their interactions (*P* < 0.001) because cumulative germination percentage of the nontreated seeds was significantly higher than of 12-week cold stratified ones at under the same light and temperature conditions. In addition, a three-way ANOVA showed that germination velocity was significantly affected by light (*P* < 0.001), temperature (*P* < 0.001), cold stratification (*P* < 0.001), and the interaction between light and temperature (*P* < 0.001) as well as by interaction between temperature and cold stratification (Table 1).

### 3.2. Effect of Salinity on the Germination

**Figure 3:**
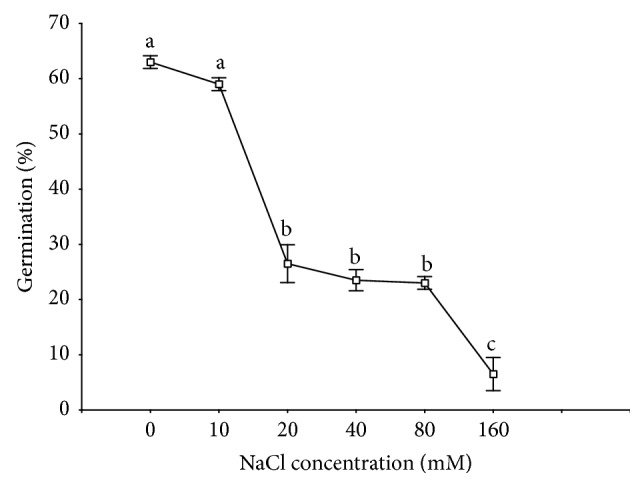
Effects of NaCl concentration on germination of* Rumex confertus* seeds (*n* = 4) incubated at 20°C with 12 h photoperiod for 20 days. Data points represent means from 4 replicates. Different letters indicate significant differences (ANOVA, Tukey's test at *P* < 0.05) in the germination percentages and rate of germination of seeds among different NaCl concentrations. Vertical bars represent standard deviations of the means.

Seed germination was higher in distilled water than in any of the saline treatments (post hoc comparison, *P* < 0.05 for the difference). Germination decreased as salinity increased (*P* < 0.05), with minimal germination at 160 mM NaCl (7%) (Figure 3). The index of germination velocity calculated by using a modified Timson's index showed that the rate decreased with an increase in salinity and the highest rate of germination occurred in distilled water.

### 3.3. Moisture Stress

**Figure 4:**
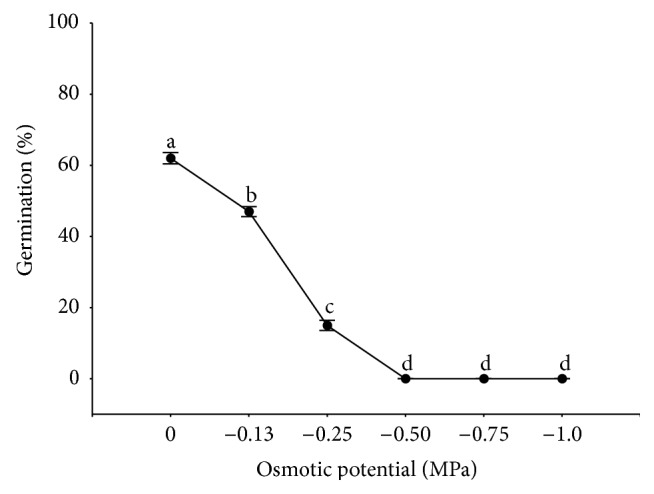
Mean germination percentages for* Rumex confertus* seeds at varying PEG-6000 solutions. Means (*n* = 4) with the same letter do not differ (*P* > 0.05). Error bars indicating standard deviation.

PEG-6000 solutions at osmotic potential −0.1 MPa to −0.5 MPa significantly inhibited germination, which was manifested in reduction of the final germination percentage. The seeds germinated in the lower water potentials such as 0 and −0.13 MPa (66 and 48%, resp.); only 16% of seed germinated at osmotic potential −0.25. PEG at −0.5, −0.75, and −1.0 MPa inhibited germination completely (Figure 4).

### 3.4. Effect of pH on Seed Germination

**Figure 5:**
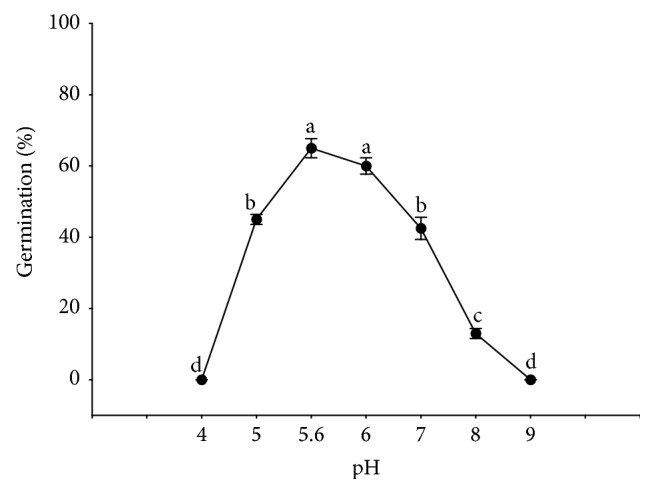
Final percent germination of* Rumex confertus* seeds as effected by pH after incubating at 22°C with 12 h photoperiod for 20 days. Means with the same letter do not differ (Tukey's test at *P* < 0.05). Vertical bars represent standard deviations of the means.

Germination of* R. confertus* seed occurred in a range from 5 to 8 of buffered pH solutions but the highest germination occurred at pH 5.6 (deionized water control) and 6. Seeds did not germinate when pH was 4 or 9 (Figure 5).

### 3.5. Effect of NO_3_^−^ on Germination and on Radicle and Hypocotyl Lengths

**Figure 6:**
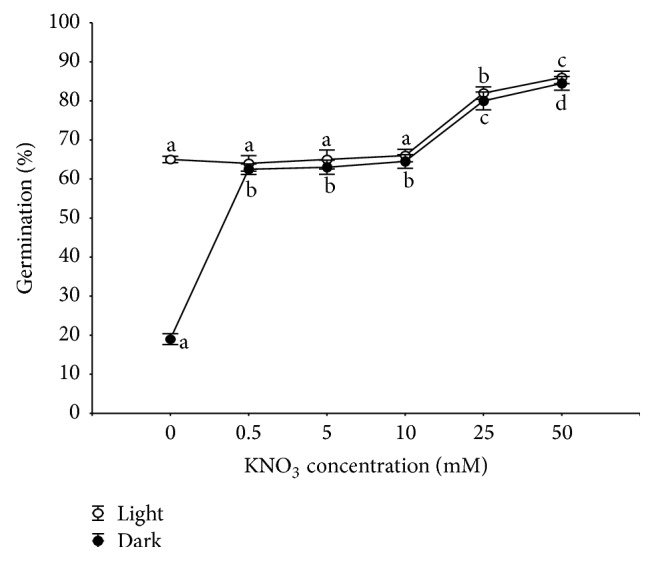
Effect of KNO_3_^−^ concentration on cumulative germination of* R. confertus* seeds at 22°C for 20 days. Means (*n* = 4) with the same letter do not differ among different KNO_3_ concentrations with the same light conditions (ANOVA Tukey's test, *P* > 0.05). Error bars indicating standard deviations are shown only where it was larger than the point size.

**Figure 7:**
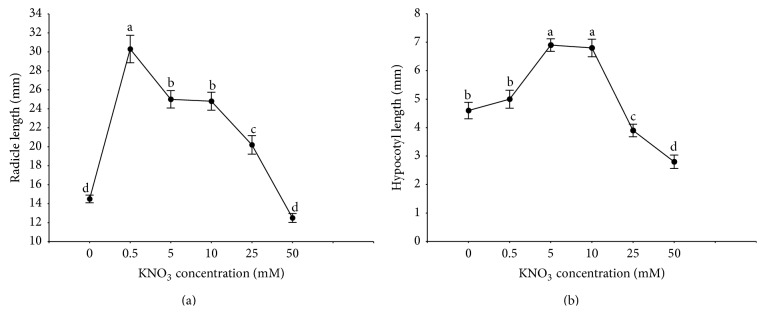
Effect of KNO_3_ concentration on the radicle (a) and hypocotyl lengths (b) of* R. confertus*. Means (*n* = 4) with the same letter do not differ (*P* > 0.05). Error bars indicating standard deviation.

The highest germination percentage (88%) was detected in light at 50 mM KNO_3_. Lower concentrations of potassium nitrate such as 0.5, 5, and 10 mM were not found to be stimulatory (post hoc comparison, *P* > 0.05). The germination percentage in the N-free control treatment under the same photoperiod was 43%. In darkness, exogenous application of KNO_3_ at all concentrations significantly increased germination of* R. confertus* (post hoc comparison, *P* < 0.05) and the highest concentration (50 mM KNO_3_) was the most effective with 83% of seed germination (Figure 6). Among the seeds incubated in light the germination rate was highest (67) at 50 mM KNO_3_ concentration followed by 25 mM when the germination rate was 64. The germination rates at 0.5, 5, and 10 mM KNO_3_ concentrations were similar to the control. In darkness, the influence of pretreatment with the solutions of KNO_3_ on the rate of germination was significant (post hoc comparison *P* < 0.001). The highest germination rate (66) was observed at 25 and 50 mM concentrations. KNO_3_ applied at concentrations of 0.5, 5, and 10 mM caused moderate promotion of seed germination, that is, 43, 48, and 50, respectively. When germination percentages and rate of germination were analysed significant effects (*P* < 0.001) were found for light, nitrate, and their interaction (Table 2). Furthermore, different concentrations of KNO_3_ produced variable results for radicle and hypocotyl lengths. The radicles from the control seedlings (the N-free treatment) were the shortest, whereas those under the lowest and moderate KNO_3_ concentrations were the longest, but no significant difference (*P* > 0.05) between 50 mM KNO_3_ and the control was observed (Figure 7(a)). Increasing KNO_3_ concentration resulted in an increase in the hypocotyl length, but no significant difference (*P* < 0.05) between 0.5 mM KNO_3_ and the control was observed. The hypocotyl length was noticeably inhibited by 25 and 50 mM KNO_3_ concentrations (Figure 7(b)).

### 3.6. Effect of Sand Burial Depth on Seedling Emergence

We found that sand burial depth had significant effect on* R. confertus* seedling emergence. Only from the seeds buried at the depth of 0.5 cm, 15% seedling emergence was observed. At the depths > 0.5 cm, the percentage of seedling emergence was almost zero.

## 4. Discussion

Freshly matured seeds of* R. confertus* are nondormant and high percentages of germination were obtained when the seeds were incubated in light at 22°C. Other* Rumex*-species seeds are also non dormant at maturity [8]. Stosik [21] showed the results comparable to our light Experiment 1 under the same temperature conditions (22°C). However, our results are contrary to those reported by Almazova and Rabornov [19], who stated that 90–98% of seeds germinated on flood-plain meadows of the river Oka in central Russia. These differences may result from longer time of seed maturation of genetic variants of* R. confertus* from various geographic locations. Variability in the germination response of seeds from different origins is well known and was previously reported for* Erica* species ([34] and the references therein) and* Impatiens* species [4]; it may be interpreted as evolutionary adaptation to climate heterogeneity.

Light is one of the environmental factors that may regulate seed germination and early establishment of seedlings because phytochrome-mediated responses play a critical role in determining the time of germination and thus become a crucial part of the evolutionary strategy to impose conditional dormancy to protect seedlings from environmental extremes [2, 35]. In general, small-seeded species are more likely to have a light requirement for germination than species with larger seeds [13].* R. confertus* has fairly small seeds (mean seed mass = 0.017 mg) that are light sensitive. The germination percentage and rate of germination were significantly higher in light than in darkness at all temperatures tested. This agrees with the work done on* R. crispus* by Samimy and Khan [36] who reported that the seeds failed to germinate in the dark, but germinated readily in light at 15, 20, and 25°C. For* R. confertus*, light-sensitivity results differed between our study and Jehlik et al. [26] who recorded that germination of* R. confertus* seeds in darkness was slightly slower but finally there was no significant difference between light and dark treatments. This difference may be explained by the fact that in the latter studies exposure to light during the counting period may have been sufficient to promote germination. In the current study, the differences in results between the tested light conditions cannot be fully explained because of limitations in the experimental design. As regards the light, it is abundant only on the soil surface and physiologically active light flux rarely penetrates more than a few mm into soil [10]. Seeds germinate successfully on or near the soil surface when temperature and edaphic conditions are favourable. If buried at soil depths to which light cannot penetrate, they may become part of a persistent soil seed bank. The light prerequisite for germination in* R. confertus* collected from meadows of the Warta River valley may reflect ecological strategy for survival in the very competitive environment. High and rapid germination under full light partially explains the success of* R. confertus* in colonising vegetation gaps or open places without vegetation cover.

The conditions required for effective cold stratification in general resemble the natural conditions to which seeds are exposed in winter and early spring [37]. Totterdell and Roberts [12] argued that stratification not only resulted in dormancy release but also could induce dormancy and that these two independent processes may occur at the same time. Since seeds of* R. confertus* are nondormant at maturity, they are expected to germinate in the field directly after shedding in late summer and early autumn at high and/or moderate temperatures. However, germination stops by the end of September at the latest, when daily averages are usually still well above 10°C. The seeds that not germinated in late summer and early autumn enter dormancy during the cold autumn and winter temperatures. Induction of secondary dormancy at cold temperatures prevents the germination at disadvantageous temperatures. Due to regular periods of frost, late autumn and winter are often the most hazardous seasons for seedlings in temperate climate habitat. Spring germination, in contrast, starts no earlier than late April or May and continues over the whole summer season when exposition to light is intensive. A seedling emergence pattern very similar to that of* R. confertus* was observed for* R. obtusifolius* [12, 17] and* R. crispus* [12].

PEG preventing water uptake is being widely used to impose water stress that inhibits seed germination [38]. The effect of increased salt concentration or soil water deficit upon germination percentage and rate of seedling emergence was shown in many crops [39], where PEG was used to simulate water-stress conditions. Kaya et al. [39] observed that PEG imposed stronger stress then salt on seed germination in sunflower and they suggested an osmotic effect of PEG rather than ion accumulation, because when PEG stress was removed, the seeds were able to germinate. Rahimi and Kafi [30] studying different levels of drought stress imposed by PEG on* Portulaca oleracea* reported no significant differences in germination percentage up to the potential level of −0.75 MPa. Poor seed germination under PEG-induced water deficit was also observed in* Amaranthus caudatus* [31],* Phaseolus mungo* [40] and* R. acetosella* [7]. In contrast* R. crispus* still had 100% germinability at osmotic potential of −1.5 MPa [33]. Our results indicated that* R. confertus* seeds were not able to tolerate short-termed osmotic stress lower than −0.25 MPa. Thus,* R. confertus* spread may be restricted to moist soil due to its inability to germinate under high soil moisture conditions. We suggest that only low water potential level in the soils was beneficial for* R. confertus* seed germination and for water transport thus promoting growth and development of plants seedlings.

Concentrations and composition of salts, duration of exposition, plant type and cultivar, and environmental conditions are some of the factors that play a role in plant tolerance [41]. It is not known if salinity prevents or merely retards the germination process in* R. confertus*. Our results demonstrated that optimal germination of* R. confertus* seeds occurred under nonsaline conditions (control treatment) and germination decreased as soil salinity increased with minimal germination at 160 mM (7%), because it probably exceeded individual tolerance limits. This indicates that* R. confertus* seed can remain ungerminated on the salt-rich soil surface. Reduction in the percentage of germinating seeds induced by an increase in salinity stress was described by numerous authors (e.g., [42–44]). This can be attributed to hyper osmotic stress and/or toxic effects of Na^+^ and Cl^−^ ions, depending on a plant species. Salinity may create an external osmotic potential limiting water absorption by seeds or sodium and chloride ions may accumulate in germinating seeds, resulting in a toxic effect [45, 46].

The degree of habitat colonisation was correlated with availability of all nutrients and of specific nutrients such as phosphorus, calcium, and nitrogen (see [1] for the list and references). Some nitrates and other nitrogenous compounds are known to promote germination in a large number of plant species and they are priming agents in germination media [47–49]. Nitrate is an important nitrogen source for plants but also a signal molecule that controls various aspects of plant development. The concentration and form of nitrogen could be an important factor affecting successful of seed germination and development of plants. Lower concentrations of nitrate solutions promoted germination of* Commiphora wightii seeds*, while higher ones inhibited it [50]. Ponert et al. [51] demonstrated that germination of mature seeds and protocorm development of* Pseudorchis albida* (Orchidaceae) were inhibited by nitrate at high concentrations. These authors showed that the exact role of nitrogen in orchid seed germination still remained unclear. Other work also confirmed that nitrate prevented the onset of dormancy of* Arabidopsis* seeds they accelerated germination and development [52]. Nitrate provided exogenously or by mother plants to the produced seeds, acts as a signal molecule favouring germination in* Arabidopsis*. This signalling may involve interaction with the abscisic acid or gibberellin pathways. The positive effect of nitrate on seed germination is linked to phytochrome (Pfr) [53], because nitrate may enhance the number of Pfr-receptors [54]. For several species, significant interaction was found between light and nitrate. In some cases, nitrates were unable to stimulate seed germination in darkness (see [55] for the list and references). The positive influence of nitrogenous compounds on germination is concentration- and species-specific. Nitrates is obtained in the range of 0 to 50 mM promoted germination of species from Western Australia [56] and the Iberian Peninsula [57], but their high levels reduced germination of* A. sagittata* [58]. Different optima (high nitrate in light period; low, medium and high in darkness) may enable* R. confertus* to occupy a wide range of habitats and to cope with the remarkably variable NO_3_^−^ concentration in ruderal soil [59].

*R. confertus* germinated in a broad range of pH (5–8), indicating that the hydrogen-ion concentration of soil may not be a limiting factor for germination in most soils. Their distribution is probably dependent on a combination of several ecological factors rather than on pH alone. These results are similar to those reported for the related species* R. scutatus* [60] and* R. acetosella* [61].

Seedlings did not emerge when the seeds were buried >0.5 cm deep. A primary reason why seeds do not germinate while buried is the fact that they have a light requirement for germination. The result of an additional experiment (data not presented) indicated that 66% of* R. confertus* seeds germinated when they were sown on the sand surface. Reduced seedling emergence from seeds buried at deeper depths was also reported for other species [62, 63]. Another reason for the reduced seedling emergence seemed to be the fact that following seed germination, the emergence of seedlings depends on the seed energy reserves [64]. The emergence of seedlings is also related to the weight of seeds, hence to the amount of energy reserves within them [63]. In addition, it has been suggested that for the smallest seeded species (seed mass < 1 mg), maximal effective burial depths for emergence are <0.5 mm [65]. The small size of* R. confertus* seeds might be one of the causes of seedling emergence at depths < 0.5 cm. Another hypothesis is that higher mechanical resistance of deeper soil layer impeded early radicle growth and hypocotyl elongation and consequently reduced seedling emergence in deeper layers [62]. In addition, increased water content decreases the concentration of oxygen at the same burial depth, thus causing the resulting oxygen deficiency which may inhibit seedling elongation.

## 5. Conclusions

Our results suggest several possible mechanisms to explain the rapid expansion of* R. confertus* in central Europe. The seed germination of this species requires high moisture and low soil salinity, moderate temperatures, and presence of light. The seeds enter dormancy during the cold autumn and winter temperatures. This dormancy is broken again in a following growing season. The fact that seed germination is sensitive to light may facilitate fast germination of this weed from soil seed banks in disturbed soils of disturbed habitats (such as roadsides, railway tracks, and embankments) and overgrazed pastures in central Europe and may finally favor its colonization of newly invaded habitats. Our findings indicate that the positive effect of nitrates on seed germination explain the ability of* R. confertus* to colonize soils with high nitrogen concentration. Secondary dormancy allows* R. confertus* seeds to survive in seed banks for many years; thus, although plant control techniques for this weed can be successful, its complete eradication from meadows is difficult.

## Figures and Tables

**Table 1:** Three-way ANOVA of effects of temperature (*T*), light condition (*L*, light versus dark), cold stratification (CS, nontreated versus 12-week cold stratified) seeds of *Rumex confertus*, and their interactions on seed germination and rate of germination. Data were transformed (arcsine) to achieve normality.

Dependent variable	Factor	df	MS	*F* value	*P* value
Germination (%)	Temperature (*T*)	2	15.48	14.35	<0.001
	Light (*L*)	1	9.77	6.45	<0.001
	Cold stratification (CS)	1	17.88	13.35	<0.001
	*T* × *L *	2	1.54	16.66	<0.001
	*L* × CS	1	1.94	19.63	<0.001
	*T* × CS	2	5.250	53.23	<0.001
	*L* × *T* × CS	2	0.91	9.18	<0.001

Rate of germination	Temperature (*T*)	2	25.67	660.18	<0.001
	Light (*L*)	1	17.44	448.35	<0.001
	Cold stratification (CS)	1	15.81	406.79	<0.001
	*T* × *L *	2	0.63	16.36	<0.001
	*L* × CS	1	1.53	39.55	<0.001
	*T* × CS	2	5.36	137.99	<0.001
	*L* × *T* × CS	2	0.82	20.74	<0.001

**Table 2:** Two-way analysis of variance (ANOVA) showing the effect of particular factors (light and NO_3_^−^) on final and rate of germination. Data were transformed (arcsine) to achieve normality.

Dependent variable	Factor	df	MS	*F* value	*P* value
Germination (%)	Light	1	0.41	48.0	<0.001
	NO_3_^−^	4	0.89	103.8	<0.001
	Light × NO_3_^−^	4	0.51	60.1	<0.001

Rate of germination	Light	1	0.24	37.0	<0.001
	NO_3_^−^	4	1.33	204.2	<0.001
	Light × NO_3_^−^	4	0.61	94.2	<0.001
